# ASO Author Reflections: Navigation-Assisted Surgery for Locally Advanced and Recurrent Rectal Cancer: The NAVI-LARRC Trial

**DOI:** 10.1245/s10434-023-14058-2

**Published:** 2023-08-12

**Authors:** Arne M. Solbakken, Kjersti Flatmark

**Affiliations:** 1 Department of Gastroenterological Surgery, The Norwegian Radium Hospital, Oslo University Hospital, Oslo, Norway; 2 Institute of Clinical Medicine, University of Oslo, Oslo, Norway; 3 Department of Tumour Biology, Institute for Cancer Research, The Norwegian Radium Hospital, Oslo University Hospital, Oslo, Norway

## Past

For patients with locally advanced rectal cancer (LARC) or locally recurrent rectal cancer (LRRC), complete tumor removal with clear resection margins (R0) results in the best prognosis. However, when tumors involve multiple pelvic organs and structures, achieving R0 resection can be challenging, particularly in the posterior and lateral compartments, and the procedures carry significant risk of postoperative functional impairment. In other surgical disciplines, in which precise anatomic identification is needed, implementing navigation-assisted surgery has proved to be useful, whereas for rectal cancer, navigation has been reported in only a handful of studies, and indications remain to be defined.

## Present

In the NAVI-LARRC trial, the authors investigated the feasibility of navigation-assisted surgery for 17 highly selected LARC or LRRC patients with advanced tumors in the posterior and lateral pelvic compartments.^1^ Manual preoperative segmentation on two-dimensional MRI was found to be time-consuming, but participating surgeons reported that the resulting three-dimensional (3D) model gave an increased understanding of the spatial relations between tumor and adjacent structures. Intraoperative navigation based on optical tracking was accurate, and navigation on segmented anatomy and pelvic bone gave an increased decisiveness during dissection. In the majority of cases, R0 resection was obtained. Based on the experience gained from this trial, the authors believe navigation-assisted surgery may be useful in selected cases of LARC and LRRC.

## Future

The navigation technology is still in its infancy, and several challenges must be met before navigation can become part of the standard armamentarium for treatment of rectal cancer. Although additional screens in the console monitor allow visualization of 3D preoperative images alongside the view of the operative field in robot-assisted surgery and holographic headsets might soon enable overlay of 3D preoperative images with patient anatomy in the surgical field during open surgery,^2,3^ the segmentation process needed for creating the images is still laborious. Numerous computer segmentation programs are available, and many allow automated segmentation of pelvic bone as well as semi-automated segmentation of organs with homogeneous radiologic signal, but the tumor and less prominent soft tissue structures still have to be segmented manually to secure precise delineation.^4^ Deep learning and artificial intelligence might facilitate this, but currently, the tedious segmentation process hinders routine pre- and intraoperative use of 3D imaging.

For intraoperative navigation, the positional shift of soft tissue structures as dissection proceeds is still a challenge. Although the pelvic bone remains in place until resected, segmented soft tissue structures are displaced from their original position upon dissection, leaving the “map” in disagreement with the “ground,” and navigation will no longer be accurate. Solutions to this challenge have been sought,^5^ but to the authors’ knowledge, no currently available technology can effectively adjust segmentation boundaries to compensate for movement of multiple pelvic structures in real time. Failure to solve this issue might limit intraoperative navigation for rectal cancer to selected patients with tumors and structures located close to the pelvic bone in the foreseeable future.
